# EFFECTIVENESS OF TARGETED WEB-BASED ADVERTISING WHEN RECRUITING OLDER ADULTS FOR A CLINICAL TRIAL

**DOI:** 10.1093/geroni/igae098.3031

**Published:** 2024-12-31

**Authors:** Kathryn Baldyga, Courtney Millar, Ike Iloputaife, George Taffet, Lewis Lipsitz

**Affiliations:** Hebrew SeniorLife, Roslindale, Massachusetts, United States; Hebrew SeniorLife, Roslindale, Massachusetts, United States; Hebrew SeniorLife, Roslindale, Massachusetts, United States; Houston Methodist Hospital, Houston, Texas, United States; Hebrew SeniorLife, Boston, Massachusetts, United States

## Abstract

Recruiting older adults for clinical trials can be particularly challenging. Our objective was to determine if targeted web-based advertising is an effective recruitment strategy. The STAMINA Study (Senolytics To Alleviate Mobility Issues and Neurological Impairments in Aging) is a pilot 12-week study of the effects of intermittent Quercetin and Dasatinib on cognition and mobility in older adults with mild cognitive impairment and slow gait. Individuals were initially recruited using traditional strategies such as presentations and advertisements in clinical practices, subject registries, and community sites. After 10 months of slow recruitment rates, a web-based advertising company was used to deliver targeted online study advertisements based on an individual’s browsing history. Recruitment rates were calculated based on the total number of individuals engaged in each stage of the recruitment process, divided by the number of months the strategy was utilized. Web-based advertising reached more individuals (~40 individuals/month) compared to traditional methods (~5 individuals/month). Compared to traditional methods, web-based methods had at least twice the rate of expressed interest, telephone and in-person screening completion, eligibility, and enrollment. All individuals recruited from traditional methods completed the intervention, while 3 individuals recruited from web-based advertising dropped out. Those recruited using web-based advertisements tended to be younger compared to traditional methods, but were similar in racial distribution and education. Targeted web-based advertisements may be more effective in recruiting older adults for clinical trials than traditional recruitment methods, but need further evaluation of compatible study designs, potential population bias, and cost effectiveness.

